# Surface Engineering of Fluoropolymer Films via the Attachment of Crown Ether Derivatives Based on the Combination of Radiation-Induced Graft Polymerization and the Kabachnik–Fields Reaction

**DOI:** 10.3390/polym11081337

**Published:** 2019-08-12

**Authors:** Masaaki Omichi, Shuhei Yamashita, Yamato Okura, Ryuta Ikutomo, Yuji Ueki, Noriaki Seko, Ryohei Kakuchi

**Affiliations:** 1 Department of Advanced Functional Materials Research, Takasaki Advanced Radiation Research Institute, Quantum Beam Science Research Directorate, National Institutes for Quantum and Radiological Science and Technology (QST) 1233 Watanuki-machi, Takasaki 370-1292, Gunma, Japan; 2 Division of Molecular Science, Graduate School of Science and Technology, Gunma University, 1-5-1 Tenjin-cho, Kiryu 376-8515, Gunma, Japan

**Keywords:** multi-component reaction, radiation-induced graft polymerization, crown ethers

## Abstract

In this manuscript, we present the successful attachment of crown ether moieties onto fluoropolymer surfaces, via the combination of radiation-induced graft polymerization and a subsequent surface Kabachnik–Fields three-component reaction. The obtained crown ether-tethered fluoropolymer films exhibited an ammonium cation capturing ability, owing to the host–guest interactions (i.e., hydrogen bonding) between the surface-anchored crown ethers and the guest ammonium cations.

## 1. Introduction

Since their discovery in 1967 by Pedersen [1,2], cyclic ether derivatives (i.e., crown ethers) have been an important class of molecules that can selectively capture guest molecules, especially cationic species [3,4,5,6]. Owing to their tunable and facile cation capturing abilities, crown ether derivatives have been immobilized on organic polymers, silica gels, and surfaces, to mention a few—which resulted in a wide range of functional materials with unique characteristics, such as: ion-transporting [7], enantioselective guest separation [8,9], phase-transfer catalysis [3], molecular sensing [10,11], and selective cation separation abilities [12,13]. Although crown ether-immobilized materials have exceptional properties, their synthesis remains difficult, owing to the difficulties in both surface functionalization and crown ether modification steps. Of note, crown ether derivatives exhibit a strong amphiphilic nature, and their synthesis and purification steps are tedious. Therefore, the difficulties with synthesizing crown ethers suppress the attachment of the linking functionality required for surface anchoring, and hinder the preparation of crown ether-modified materials.

Recently, our groups developed an efficient synthetic approach that can attach multiple functional molecules onto the surfaces of any organic materials [14]. The key for the successful surface decoration is the facile combination of radiation-induced graft-polymerization techniques, and the subsequent surface multi-component reactions (MCRs) [15,16,17,18,19]. Among the MCRs available for synthetic polymer chemistry [20,21,22,23,24,25,26,27,28,29], we used the Kabachnik–Fields three-component reaction (KF-3CR) between aldehydes, amines, and phosphites [30,31,32]. Specifically, we discovered that the KF-3CR is compatible with polymer synthesis, both in solution and on materials’ surfaces. To demonstrate this, in our previous study, polyethylene and cellulose fabrics were subjected to radiation-induced graft polymerization of vinyl monomers containing aldehyde moieties, in order to produce fabrics with surface aldehyde groups. Then, the obtained materials containing surface aldehyde groups were subjected to the surface KF-3CR with amines and phosphites, to generate α-amino phosphonate tethered organic materials [14]. Thus, this process realized not only surface decoration with the desired functional molecules, but also the concurrent construction of α-amino phosphonate moieties that are otherwise difficult to produce. Here, we simultaneously constructed α-amino phosphonate moieties during the processes. Because crown ethers with heteroatoms differ from their corresponding normal crown ethers, they were classified as hetero-crown ethers (e.g., aza-crown ethers for N-containing crown ethers, and thia-crown ethers for S-containing crown ethers). The KF-3CR is an ideal synthetic protocol for crown ether immobilization, because it can result in the successful surface chemistry and the simultaneous attachment of heteroatoms into the crown ether moieties, which may alter their properties.

**Scheme 1:**
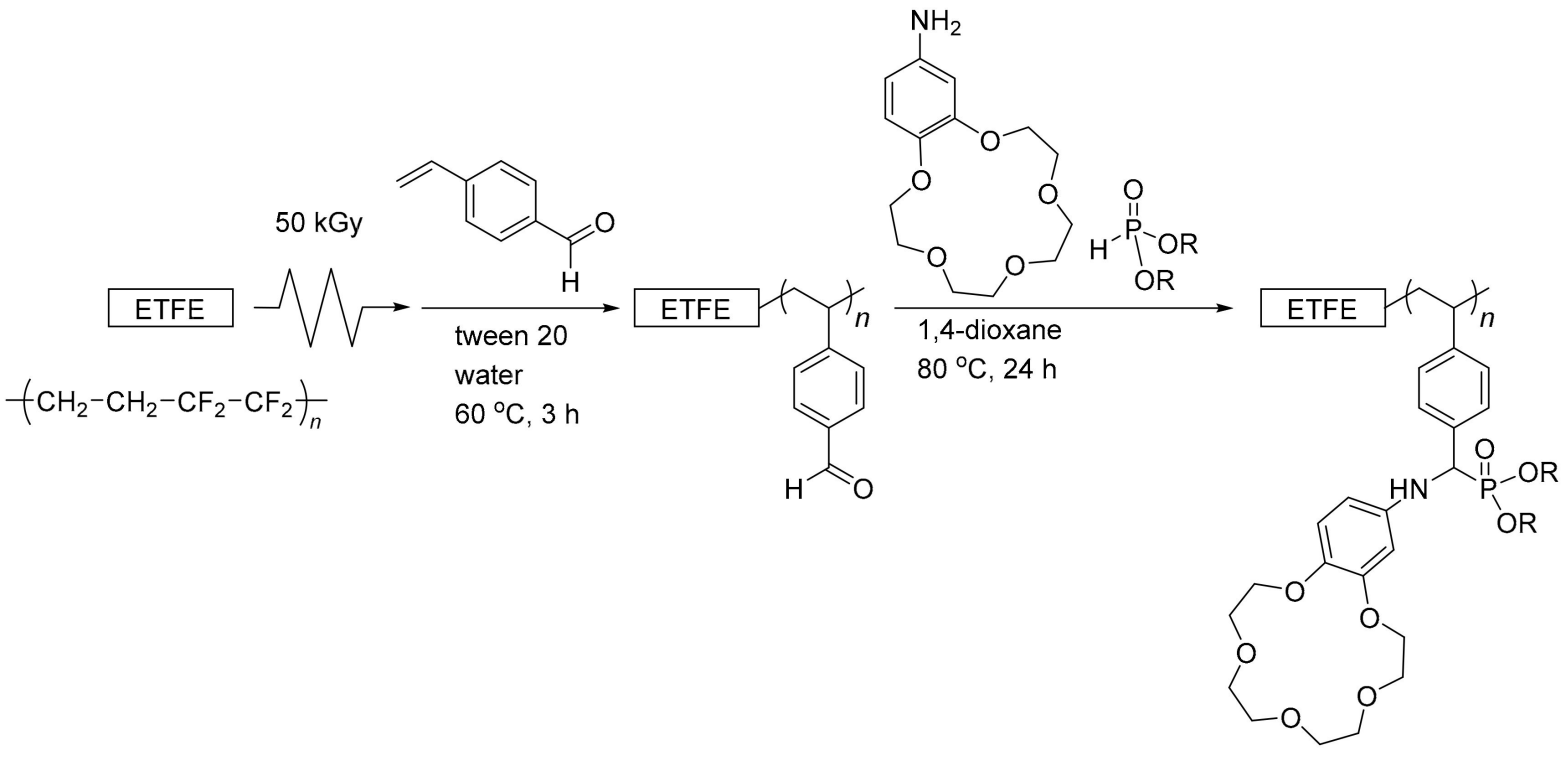
Schematic of the radiation-induced graft polymerization of St-CHO and the solid-state Kabachnik–Fields three-component reaction (KF-3CR) with amine and phosphite.

In this study, we fabricated a new crown ether-tethered organic material via the successful combination of radiation-induced graft polymerization and surface KF-3CR. To utilize radiation-induced graft polymerization, we selected a commercially available poly(ethylene-*co*-tetrafluoroethylene) (ETFE) film, because it is indispensable in modern materials science owing to its unique properties. However, typically, organic transformation reactions do not occur on the polymer backbones of fluoropolymers, owing to the lack of reactive sites and strong fluorophilic characteristics. Therefore, surface modification of fluoro-containing polymers with desired functionalities should result in unique organic materials. In this context, we turned our attentions to the radiation-induced graft polymerization technique, as the radiation exposure induces radicals on ETFE films that can allow for further chemical modifications without pre-treatment of the material surfaces, whereas; other surface initiating polymerizations—such as the surface initiating atom transfer radical polymerization—require pre-installation of the initiating functionality, prior to the graft polymerization step. In summary, we will achieve: 1) radiation-induced graft polymerization of 4-vinyl benzaldehyde (St-CHO) on ETFE fabrics; 2) surface KF-3CR of the aldehyde-grafted ETFE films with crown ethers, to generate ETFE films containing crown ethers with hetero-atom tethers; and 3) preliminary insights into the hydrogen-bonding nature of crown ether-tethered films (Scheme 1).

## 2. Materials and Methods

### 2.1. Materials

A 50-μm-thick ETFE film was purchased from Asahi Glass Co., Ltd. (Osaka, Japan), and used as a substrate. Methanol and 1,4-dioxane (super dehydrated) were purchased from FUJIFILM Wako Pure Chemical Co., Ltd. (Osaka, Japan), and used as received. Polyoxyethylene sorbitan monolaurate (Tween 20) was purchased from Kanto Chemical Co., Ltd. (Tokyo, Japan); Diisopropyl phosphite, 4′-Aminobenzo-15-crown-5-ether, and a 4-aminoazobenzene hydrochloride salt were purchased from Tokyo Chemical Industry Co., Ltd. (Tokyo, Japan), and used as received. The monomer St-CHO was prepared according to previous papers [30,33].

### 2.2. Characterization

Fourier transform infrared (FTIR) spectra were recorded in the range of 400–4000 cm^−1^ on a Perkin-Elmer FTIR spectrometer (Perkin-Elmer Frontier, Perkin-Elmer Japan Co., Ltd., Yokohama, Japan) equipped with an attenuated total reflectance (ATR) unit. A PHI 5000 VersaProbe II (ULVAC-PHI Inc., Kanagawa, Japan) spectrometer was used with a monochromatic Al-Kα radiation to obtain XPS spectra. The X-ray beam was focused to a diameter of 100 μm, with the photoelectron take-off angle of 45° relative to the surface plane. Thermogravimetric analysis (TGA) was performed using a TG-DTA6200 instrument (Seiko Instruments Inc., Chiba, Japan) at a heating rate of 10 °C/min under nitrogen flow (50 mL/min).

### 2.3. Graft Polymerization of St-CHO

The ETFE film (2 × 2 (or 2 × 3) cm^2^) was placed in a gas barrier bag. After the gas barrier bag was sealed under a nitrogen atmosphere with an oxygen scavenger, the ETFE film in a gas barrier bag was irradiated with an electron beam of 2 MeV energy and 3.9 mA current, at room temperature for a total dose of 50 kGy. The irradiated ETFE film was stored in a freezer at −80 °C until use. For the emulsion graft polymerization, 40 mg of Tween 20 and 400 mg of St-CHO were dissolved in 11.56 mL of water and stirred at room temperature for 30 min. The irradiated ETFE film was then placed in a Schlenk tube and degassed and purged with argon gas. Then, the turbid solution was degassed by bubbling nitrogen for 10 min to remove oxygen, and then added to the Schlenk tube. The mixture was heated at 60 °C for 1–3 h under an argon atmosphere. The obtained film was washed several times with water, 1,4-dioxane, and MeOH, successively, and then dried under a vacuum at 80 °C overnight, to afford the poly(St-CHO)-grafted ETFE film (ETFE-*g*-PSt-CHO).

### 2.4. Surface KF-3CR of ETFE-g-PSt-CHO

The surface KF-3CR was conducted according to our previous paper [14]. Diisopropyl phosphite (0.5 mL, 3.1 mmol) and 4′-aminobenzo-15-crown-5-ether (40 mg, 0.14 mmol) were dissolved in 3.5 mL of 1,4-dioxane. The ETFE-*g*-PSt-CHO film was immersed in the solution and heated at 80 °C for 24 h. The obtained film was washed several times with water, 1,4-dioxane and MeOH, successively, and then dried under a vacuum at 80 °C overnight, to afford the crown ether-tethered ETFE (ETFE-*g*-PAP-15-C-5-Ar).

### 2.5. Equations for the Determination of Grafting Degree (GD) Values

For the graft-polymerization steps, the GD values were determined using the following equation.

GD (%) = (*W*_g_ − *W*_0_)/*W*_0_ × 100 (1)

where *W*_0_ and *W*_g_ are the weights of the films before and after the emulsion graft polymerization, respectively.

### 2.6. Surface Host–Guest Interaction of the Crown Ether-Tethered ETFE Films

The host–guest interaction of ETFE-*g*-PAP-15-C-5-Ar with a 4-aminoazobenzene hydrochloride salt (azo-NH_3_Cl) was evaluated using the batch adsorption test. For the batch test, a 1-cm diameter sample of ETFE-g-PAP-15-C-5-Ar was prepared, and the film was immersed in 2 mL of a 5 μg·mL^−1^ azo-NH_3_Cl aqueous solution for 1 h at room temperature. After immersion in the solution, the films were washed with water and dried with nitrogen gas. The concentration of azo-NH_3_Cl in the solution was measured by using a UV–VIS spectrophotometer (U-3310, Hitachi, Ltd., Tokyo, Japan). 

## 3. Results and Discussion

### 3.1. Radiation-Induced Graft Polymerization of St-CHO

In this study, the first step is the radiation-induced graft polymerization of St-CHO of the ETFE film. In our previous report, the aldehyde-containing monomer was graft-polymerized in organic solvents such as 1,4-dioxane and MeOH. However, it is known that emulsion graft polymerization reduces the monomer concentration. Because an ETFE film with lower GD values was required in this study (see below for details), emulsion conditions were selected for the St-CHO graft polymerization of the ETFE film.

**Figure 1:**
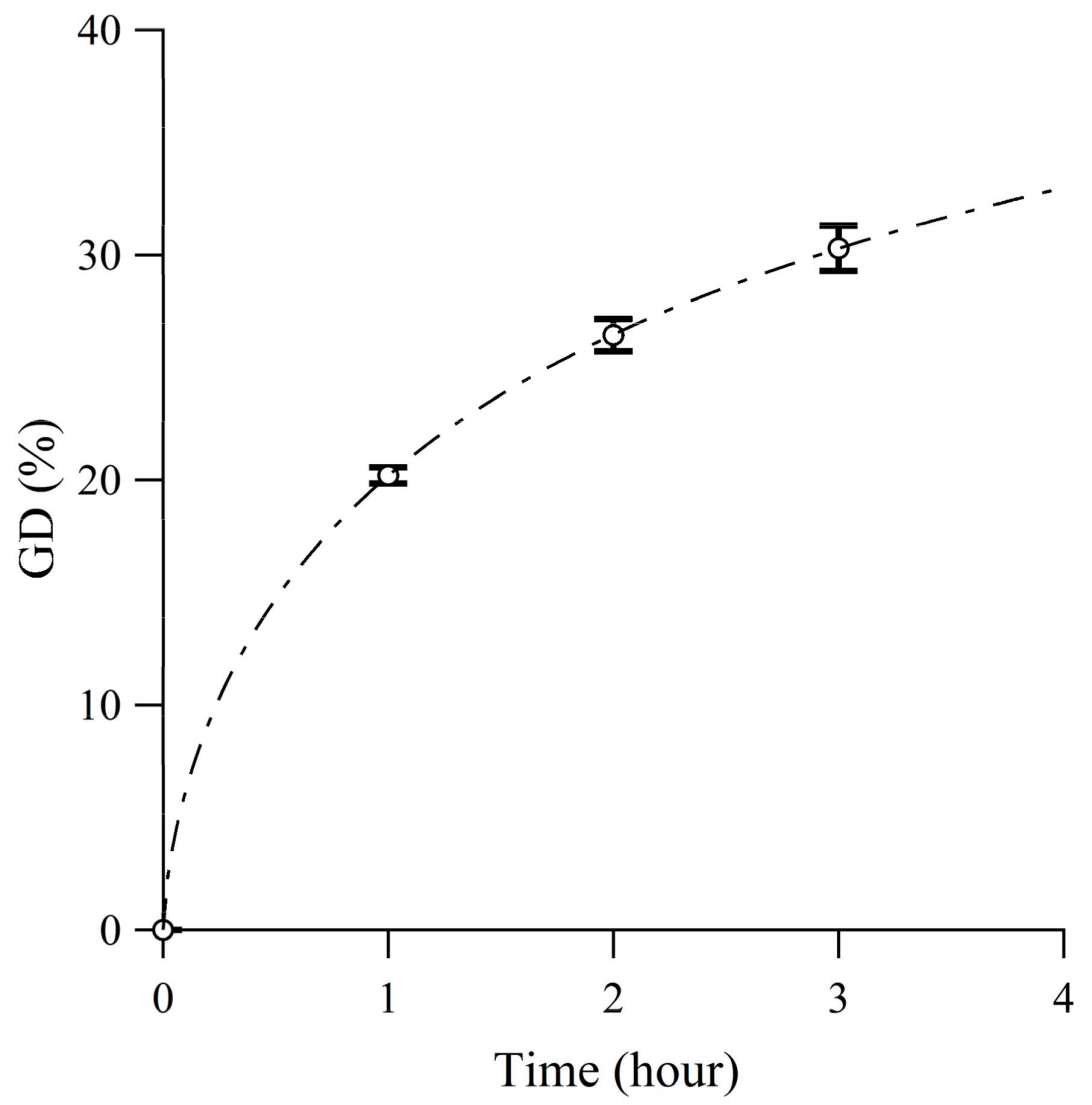
Effect of polymerization time on the grafting degree (GD), for the graft polymerization of St-CHO on the pre-irradiated poly(ethylene-*co*-tetrafluoroethylene) (ETFE) film (lines are inserted for guidance, and bars indicate standard errors calculated for three experiments for each point).

**Figure 2:**
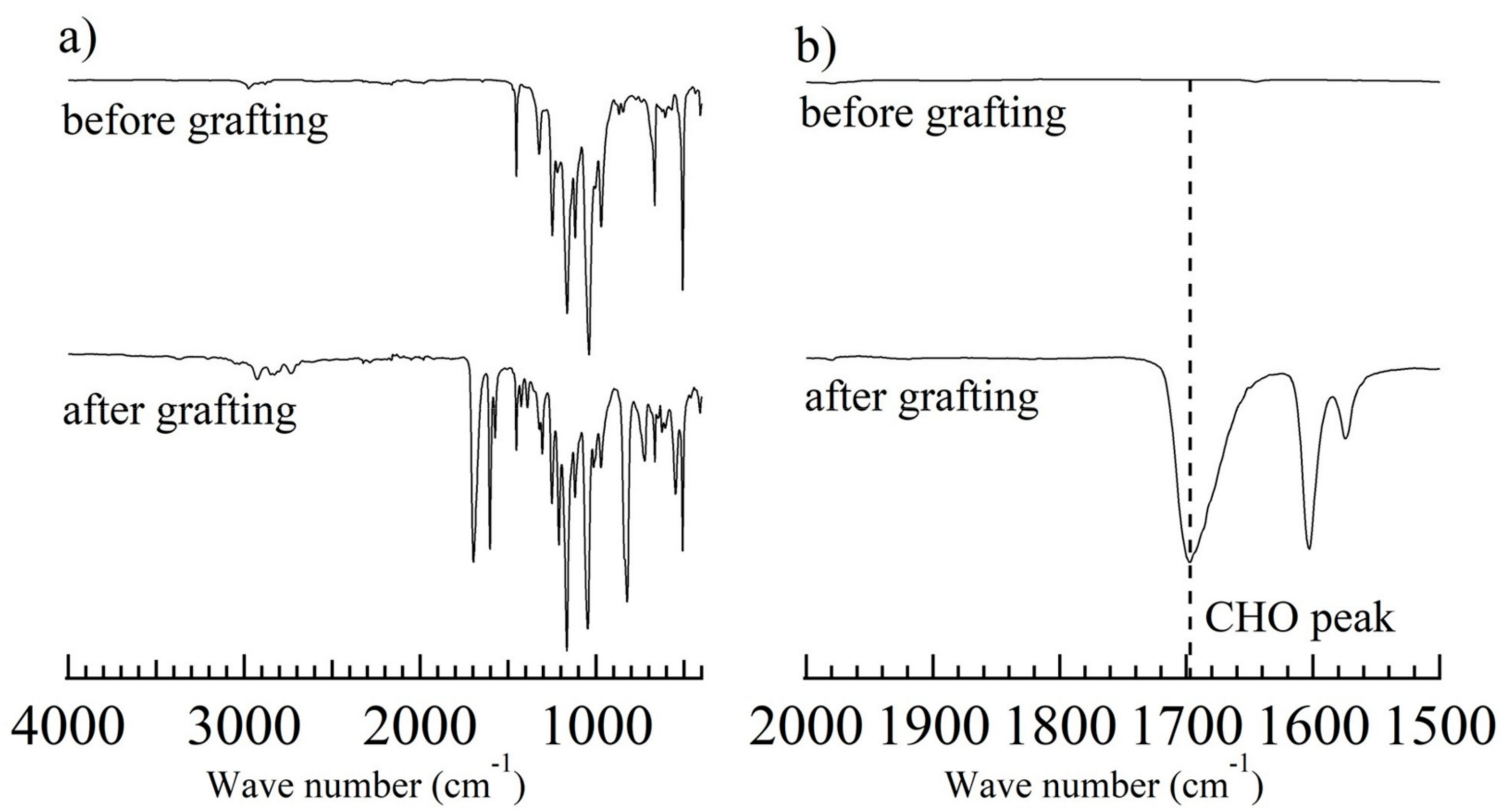
ATR-FTIR spectra (**a**) 400–4000 cm^−1^, and (**b**) 1500–2000 cm^−1^ of the ETFE film and ETFE-*g*-PSt-CHO (grafting time: 3 h).

**Figure 3:**
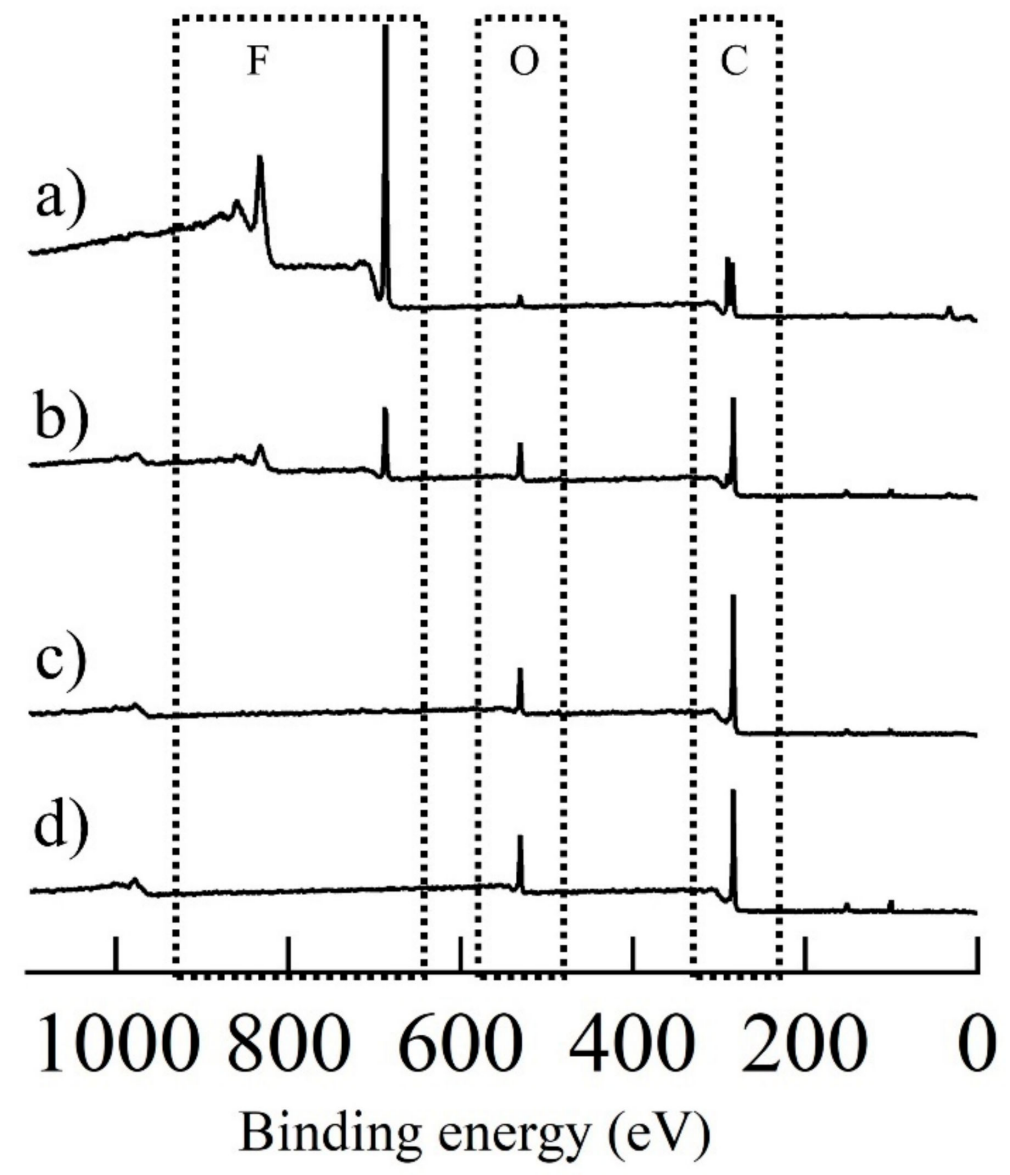
XPS spectra of the (**a**) ETFE film, and the ETFE-*g*-PSt-CHO (grafting time for: (**b**) 1 h, (**c**) 2 h, and (**d**) 3 h).

Prior to the emulsion graft polymerization of St-CHO, an appropriate surfactant was chosen. Owing to the chemical structure of St-CHO, it was immiscible with water even at low concentrations. However, the addition of a Tween 20 surfactant resulted in a turbid mixture of St-CHO and water after stirring for 30 min, which clearly demonstrated that St-CHO was emulsified in water by the Tween 20 surfactant. Therefore, the ETFE films were pre-irradiated with a dose of 50 kGy and immersed in a 3.3 wt% St-CHO emulsion at 60 °C for 1–3 h. For example, for the emulsion graft polymerization of St-CHO at 60 °C for 3 h of the pre-irradiated ETFE film (50 kGy), the GD value was 30.3 ± 1.0% (Figure 1). The facile attachment of the graft chain composed of PSt-CHO was confirmed by ATR-FTIR measurements. Figure 2 presents the ATR-FTIR spectra of the ETFE films before and after graft polymerization of St-CHO, demonstrating that the ETFE film was covered with the grafted PSt-CHO segment (ETFE-*g*-PSt-CHO). The spectrum of ETFE-*g*-PSt-CHO demonstrates a distinct C=O band at 1698 cm^−1^, which strongly suggests the successful graft polymerization of St-CHO onto the ETFE film. Owing to the successful graft polymerization of St-CHO on the ETFE surfaces, we performed surface elemental mapping. Usually, fluorine-containing polymers exhibit exceptionally strong hydrophobic and lipophobic characteristics. This unique property may result in unusual host–guest interactions on materials′ surfaces, because this surface characteristic may prevent guest cations from approaching the host crown ethers. Therefore, surface compositions were characterized using XPS measurements, as depicted in Figure 3. First, the surface of the original ETFE film exhibited peaks attributed to fluorine and carbon atoms, which correspond to the chemical structures of ETFE composed of carbon and fluorine atoms. However, the obtained ETFE-*g*-PSt-CHO film surface exhibited peaks attributed to carbon and oxygen atoms, and the peak attributed to the fluorine atom practically disappeared after 3 h, whereas these peaks still remained after the 1 h grafting. This demonstrated that the surface of the grafted ETFE film was covered by the PSt-CHO segments. The main purpose of this study is to attach a crown ether cavity onto the ETFE surface and to study its surface host–guest interactions with the guest molecules. To achieve these objectives, the modified surface must be free from fluorophilic moieties that may interfere with the approach of guest molecules to the crown ethers. Overall, we determined that approximately 30% GD leads to complete coverage of the ETFE surface layer by grafted PSt-CHO segments.

### 3.2. Attachment of Crown Ether Moieties on Materials Surfaces via the Surface KF-3CR on the ETFE Films Featuring PSt-CHO Grafts

**Figure 4:**
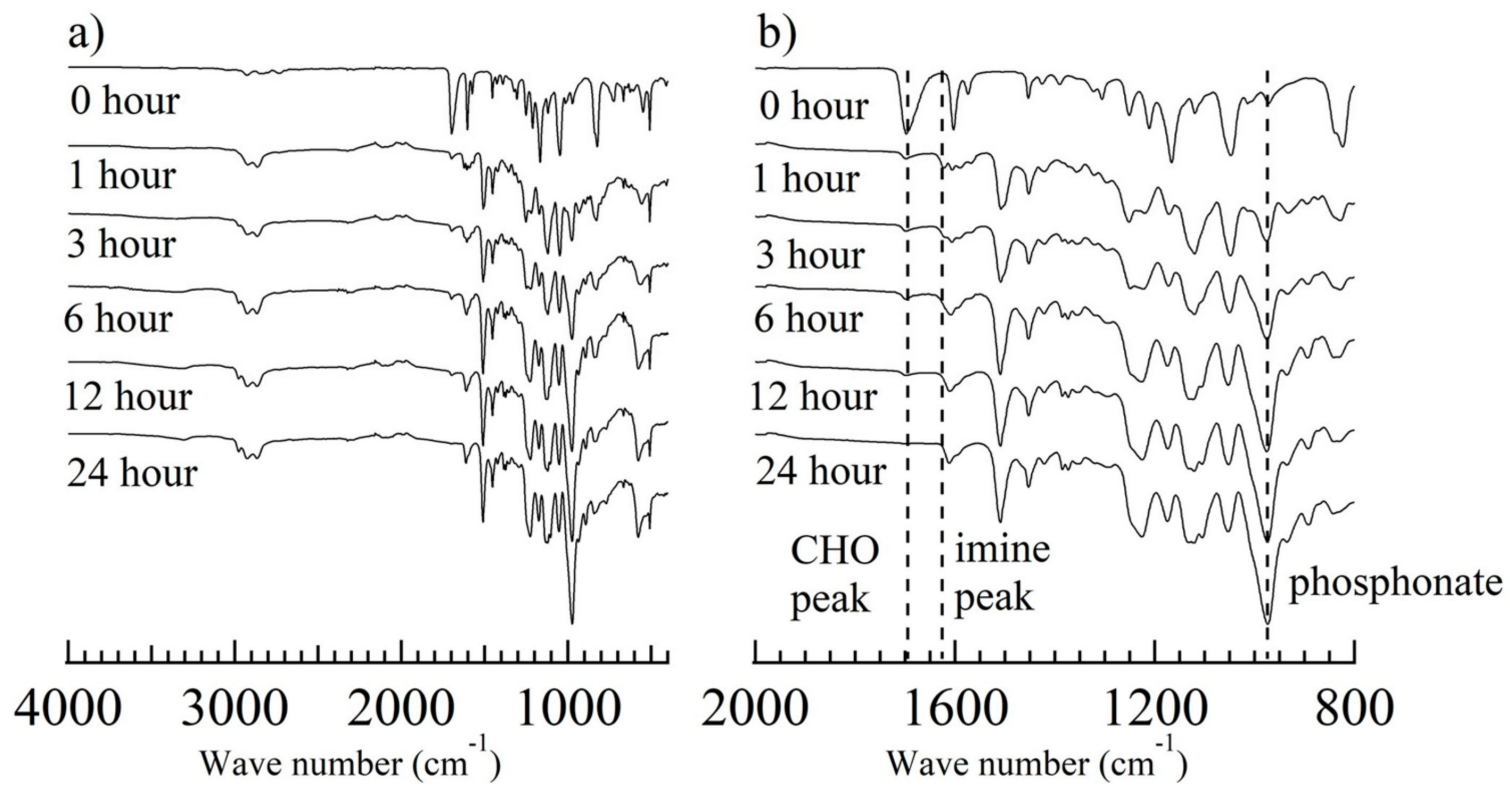
ATR-FTIR spectra ((**a**) 400–4000 cm^−1^, and (**b**) 800–2000 cm^−1^) of the ETFE-*g*-PSt-CHO (grafting time: 3 h) before and after the KF-3CR with 15-C-5-Ar-NH_2_ and diisopropyl phosphite.

**Figure 5:**
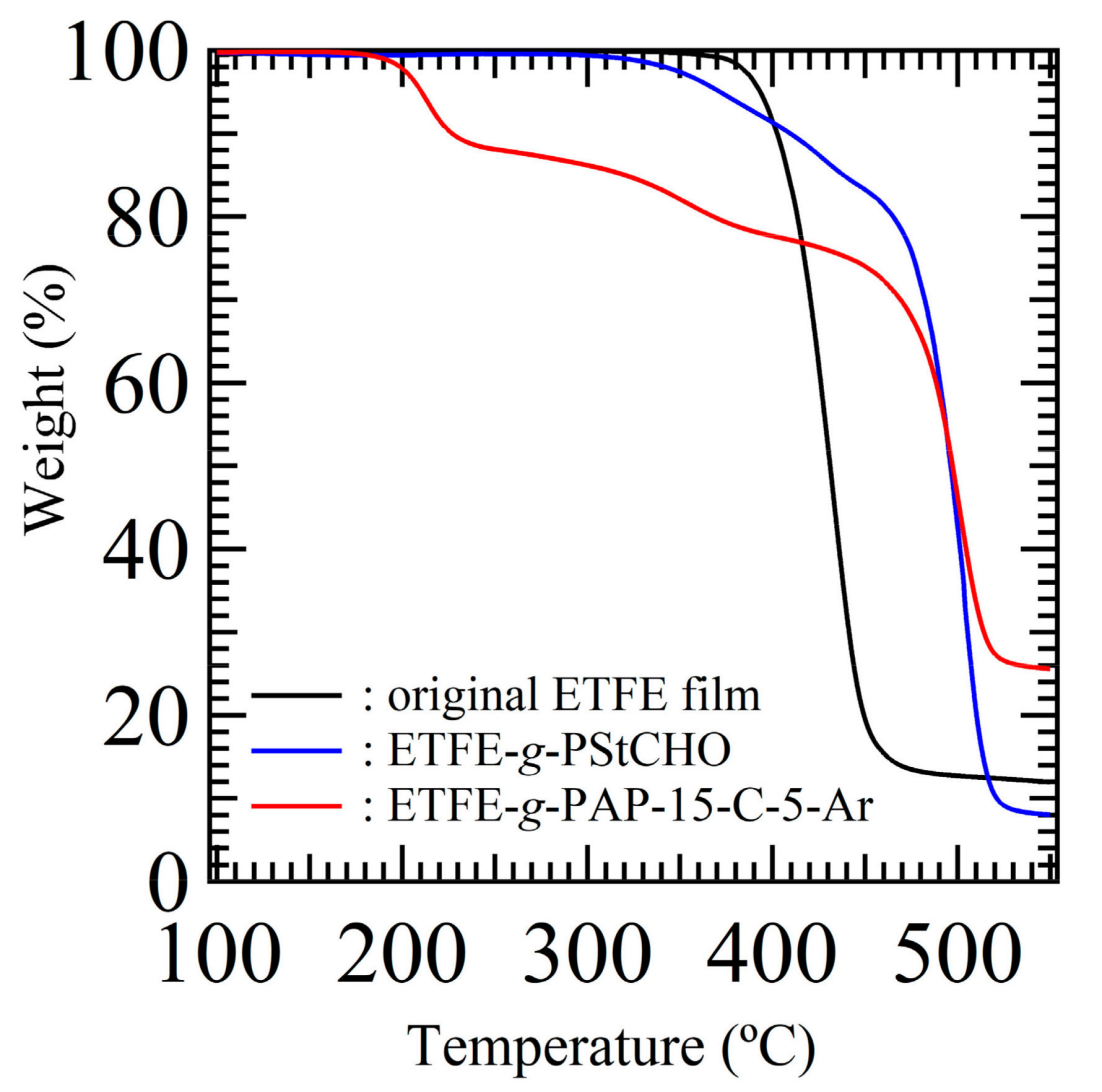
Thermogravimetry-differential thermal analysis (TG-DTA) profiles of the original ETFE film, the ETFE-*g*-PSt-CHO, and ETFE-*g*-PAP-15-C-5-Ar.

**Figure 6:**
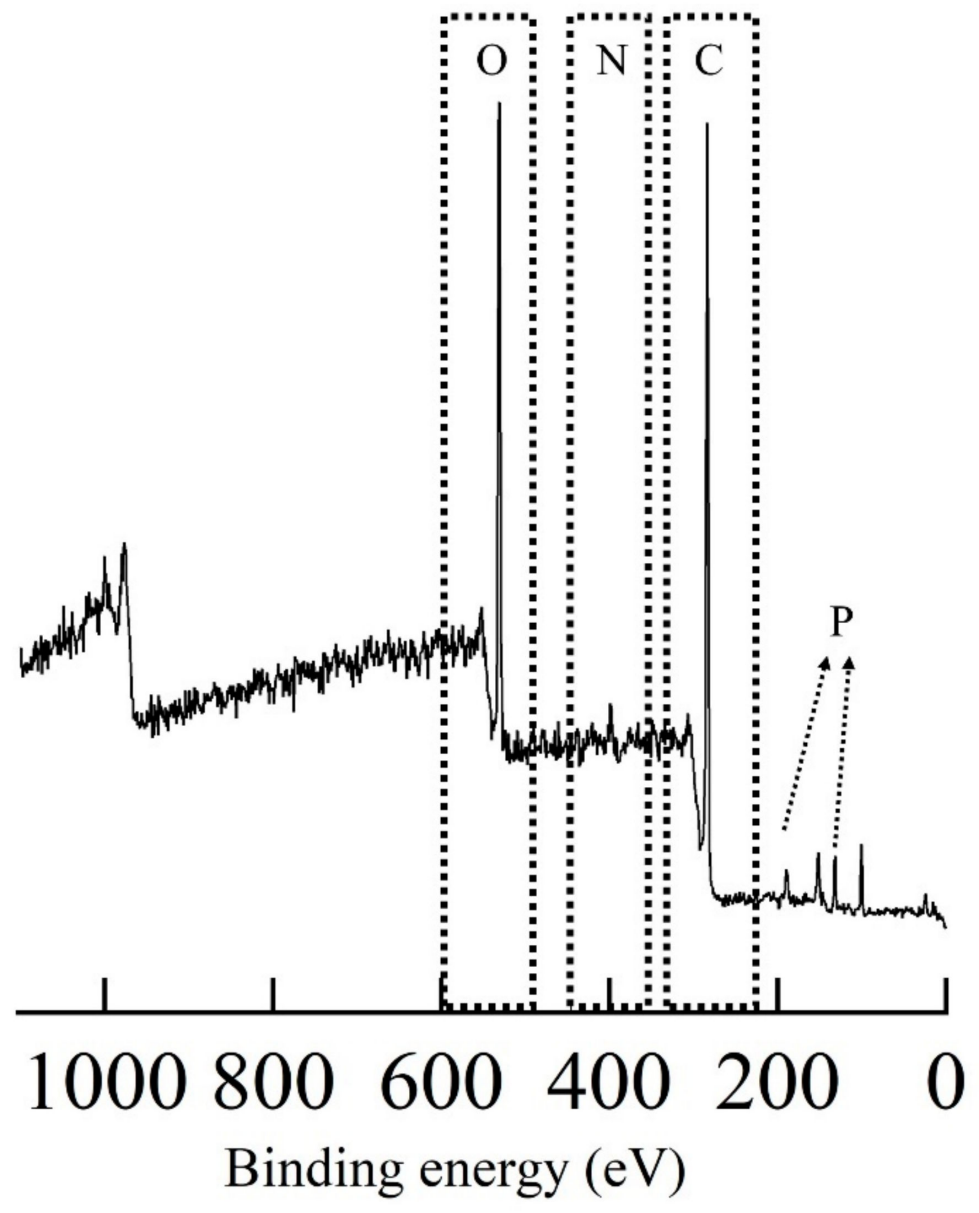
XPS spectrum of the ETFE-g-PAP-15-C-5-Ar.

Because graft polymerization of St-CHO on ETFE films was achieved, the surface KF-3CR was carried out on the ETFE-*g*-PSt-CHO to attach crown ether moieties. Thus, we selected commercially available 4′-aminobenzo-15-crown-5-ether (15-C-5-Ar-NH_2_). In a procedure similar to our previous paper [30], the surface KF-3CR was conducted with ETFE-*g*-PSt-CHO, 15-C-5-Ar-NH_2_, and diisopropyl phosphite. Specifically, ETFE-*g*-PSt-CHO was immersed in a 1,4-dioxane solution of 15-C-5-Ar-NH_2_ and diisopropyl phosphite, and the reaction progress was monitored by FTIR measurements at given times (Figure 4). In a similar manner to our previous paper, the peak at 1698 cm^−1^, which is attributed to PSt-CHO aldehydes, disappeared shortly after the reaction was initiated. However, the imine peak at 1622 cm^−1^ was observed within 1 h, and a new band developed at approximately 975 cm^−1^, which is attributed to the phosphonate moieties. After 24 h, the aldehyde and imine peaks disappeared in the FTIR spectra, proving that the amine-tethered crown ether was successfully attached via the surface KF-3CR process. The thermogravimetry-differential thermal analysis (TG-DTA) profiles of the original ETFE film, ETFE-*g*-PSt-CHO, and the obtained films, further support the successful attachment of the α-amino phosphonate ester moieties, as depicted in Figure 5. As previously reported [14,32], polymeric α-amino phosphonate esters exhibit a specific initial decomposition behavior at approximately 200 °C, probably owing to the cleavage of phosphonate ester units [34]. As depicted in Figure 5, the obtained crown ether-tethered film showed the above-mentioned initial decomposition behavior at approximately 200 °C, and increased the remaining weight at 550 °C, as compared to the corresponding original ETFE and ETFE-*g*-PSt-CHO films. These results further supported the installation of P and N heteroatoms, which thus demonstrates the successful KF-3CR. In addition, the direct evidence of the surface KF-3CR was obtained by XPS measurements of the obtained film after the surface KF-3CR, as shown in Figure 6. After the successful KF-3CR of the ETFE-*g*-PSt-CHO with 15-C-5-Ar-NH_2_, distinct peaks owing to phosphorous and nitrogen developed in the XPS spectrum. In addition, a clear increase of oxygen contents was also detected, giving a good agreement with the highly oxygen-rich structure of the crown ethers. Overall, these experimental results suggest that the surface KF-3CR performed on the surface of the ETFE-*g*-PSt-CHO with 15-C-5-Ar-NH_2_ and diisopropyl phosphite, produced the crown ether-tethered ETFE (ETFE-*g*-PAP-15-C-5-Ar), which is usually difficult to modify.

### 3.3. Surface Host–Guest Interactions of ETFE-g-PAP-15-C-5-Ar via Hydrogen Bonding

**Scheme 2:**
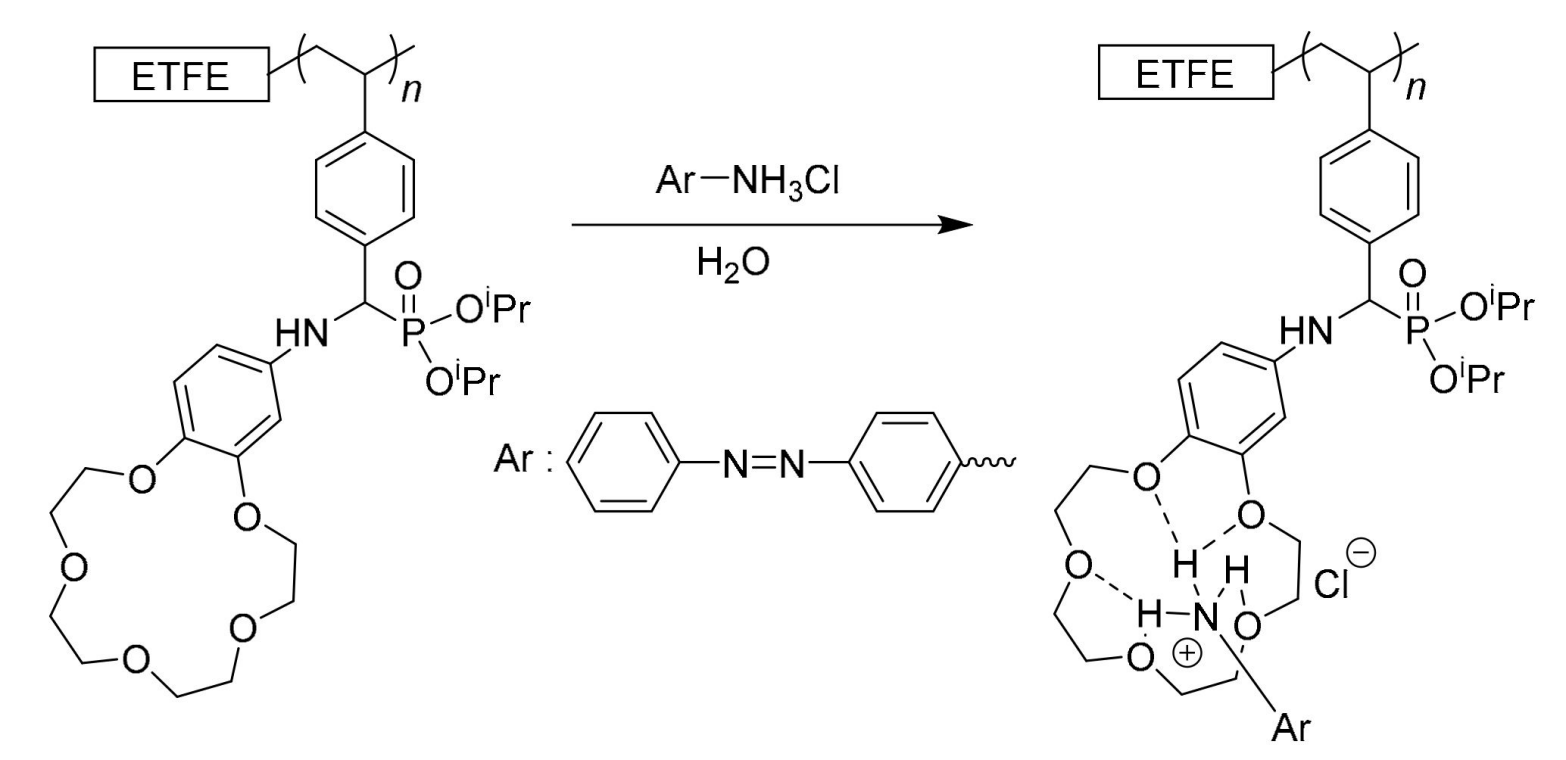
Schematic of the host–guest interaction of surface-anchored crown ethers and ammonium-based guest molecules.

**Figure 7:**
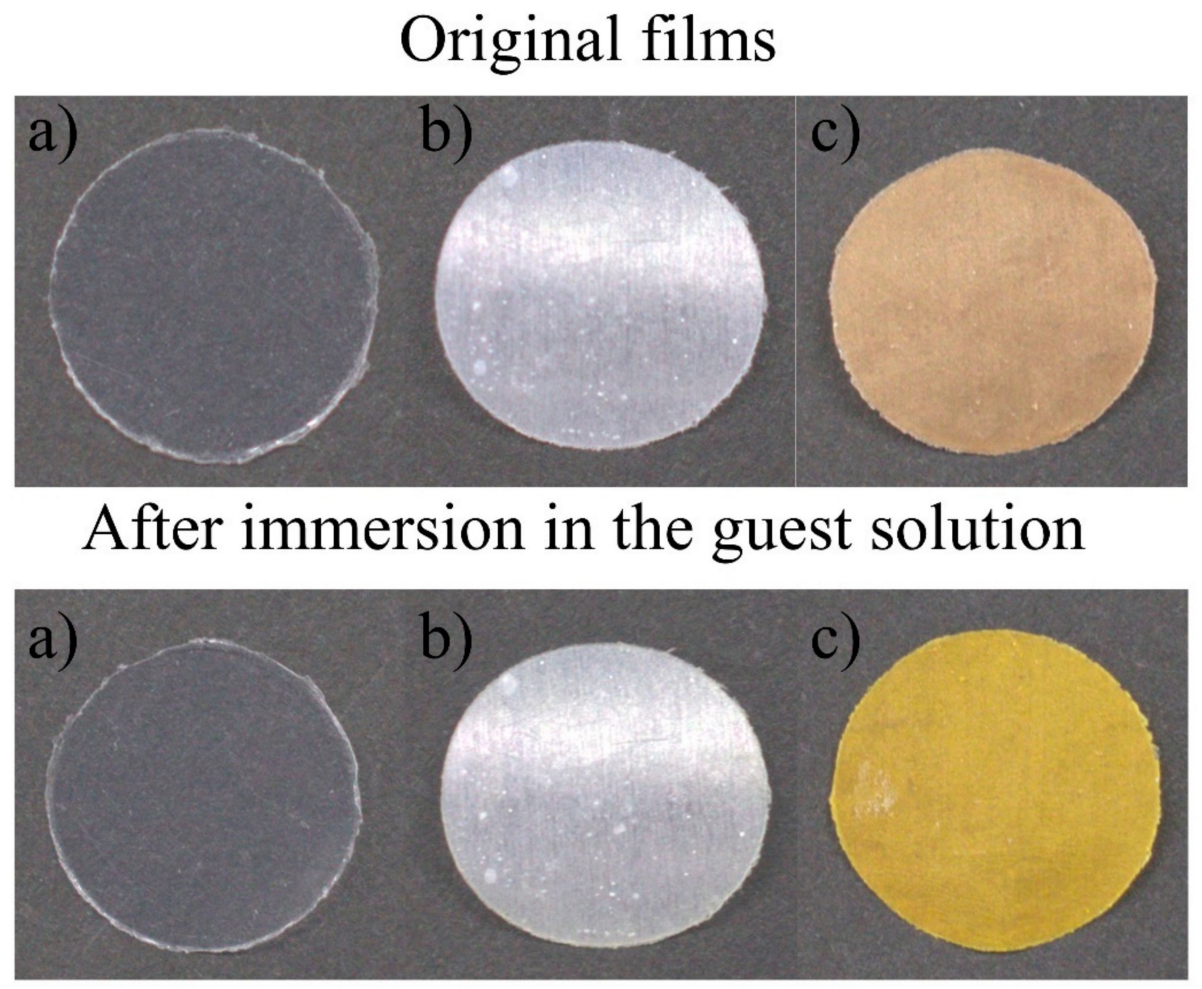
Photographs of the (**a**) original ETFE film, (**b**) the ETFE-*g*-PSt-CHO, and (**c**) the ETFE-*g*-PAP-15-C-5-Ar before (**top**) and after (**bottom**) immersion in the azo-NH_3_Cl aqueous solution.

**Figure 8:**
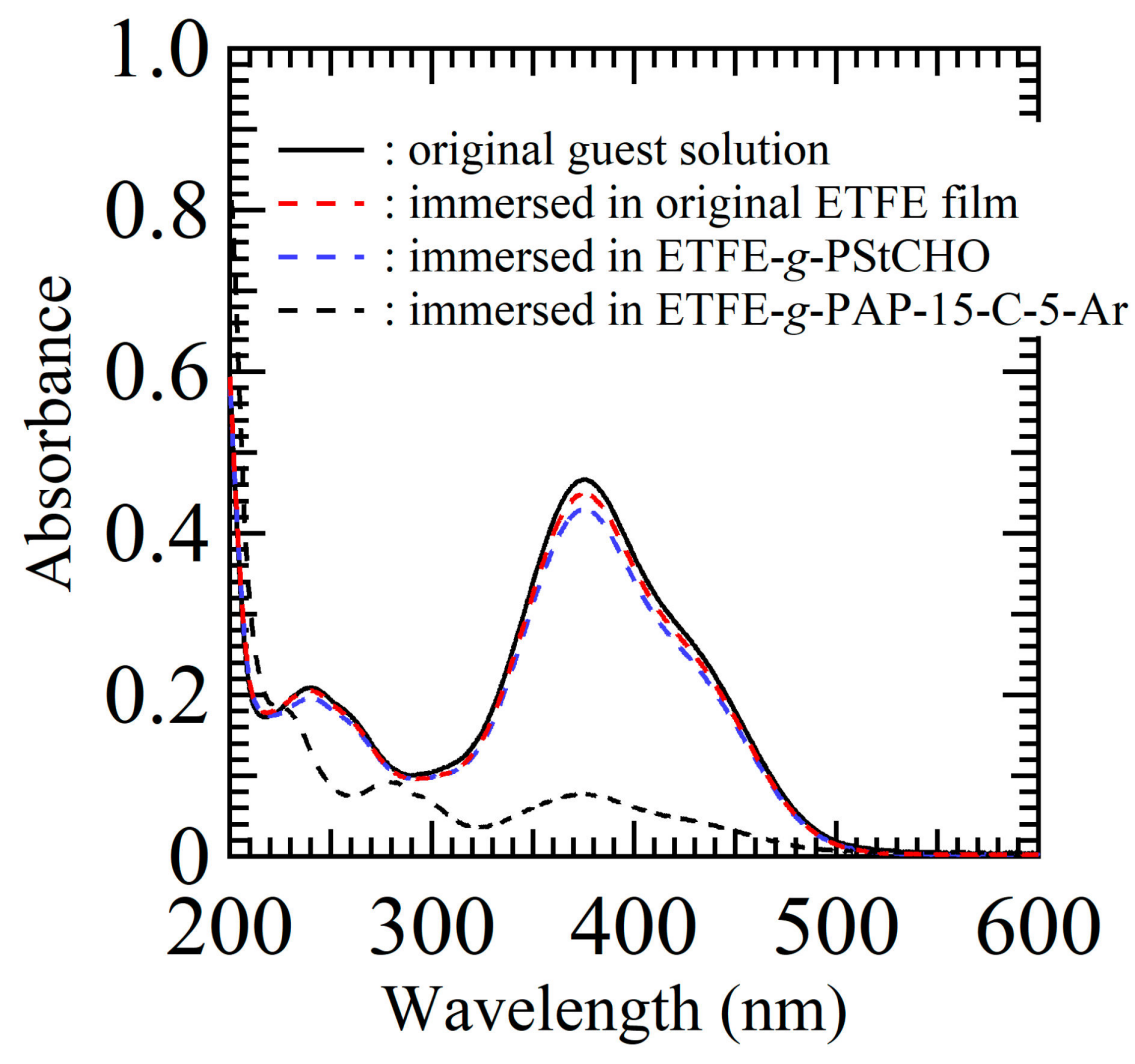
UV–Vis spectra of the aqueous azo-NH_3_Cl solution (original concentration of 5 μg·ml^−1^) before and after immersed in original ETFE film, ETFE-*g*-PSt-CHO, and ETFE-*g*-PAP-15-C-5-Ar.

The main goal of this study is to verify the host–guest interaction ability (i.e., hydrogen bonding) of surface-immobilized crown ethers. Therefore, guest-capturing via hydrogen bonding was evaluated for the ETFE-*g*-PAP-15-C-5-Ar (Scheme 2). For this, the ETFE-*g*-PAP-15-C-5-Ar film was immersed in an aqueous solution of guest ammonium salts. To visually detect the host–guest interaction, azo-benzene-based chromophores containing ammonium salts (a 4-aminoazobenzene hydrochloride salt, azo-NH_3_Cl) were selected. As designed, the chromophore was trapped on the surface of the ETFE-*g*-PAP-15-C-5-Ar film (Figure 7). Compared with the ETFE-*g*-PAP-15-C-5-Ar film, the original ETFE film did not exhibit any color change behavior upon immersion into the aqueous guest solution. In addition, the ETFE-*g*-PSt-CHO film exhibited negligible surface color changes, despite the inherent imine formation possibilities between azo-NH_3_Cl and the surface aldehydes. This, in turn, confirmed that the surface color change of ETFE-*g*-PAP-15-C-5-Ar with azo-NH_3_Cl was induced by the host–guest interaction between the crown cavity and azo-NH_3_Cl and not by imine formation with undetectable remaining aldehydes. Along with the clear color change of the material surfaces upon host–guest interaction, the azo-NH_3_Cl capturing process was precisely monitored by the UV–Vis measurements of the aqueous azo-NH_3_Cl solutions (Figure 8). The mother aqueous azo-NH_3_Cl solution concentration was adjusted to be 5 μg·ml^−1^, and the maximum absorption was observed at 376 nm. Although this peak was intact after immersion in the original ETFE film and in the ETFE-*g*-PSt-CHO, a 84% decrease in this absorption was observed when the ETFE-*g*-PAP-15-C-5-Ar film was immersed in the guest solution. This agreed with the surface color change behavior upon the immersions into the guest solution.

Overall, these results demonstrated that the crown ether moieties were successfully attached on ETFE films via the KF-3CR protocol, which yielded fluoropolymer films with guest-capturing properties.

## 4. Conclusions

In this manuscript, we have successfully attached crown ether functionalities to the surfaces of fluoropolymers that are difficult to chemically modify. Due to the facile tethering of crown ethers on the surfaces, host–guest events occurred on the surface of ETFE films. Because tethered crown ethers possess heteroatoms near the crown cavity, the proposed crown ether attachment protocol should result in the tethering of unique host molecules onto materials’ surfaces, that are expected to capture not only ammonium guests, but also a wide range of metal cations.
